# Remote binding counts: measuring distractor-response binding effects online

**DOI:** 10.1007/s00426-020-01413-1

**Published:** 2020-09-07

**Authors:** Birte Moeller, Christian Frings

**Affiliations:** Department of Psychology, Cognitive Psychology, University of Trier, 54286 Trier, Germany

## Abstract

Bindings between stimulus- and response features have received increasing attention in recent research and theorizing regarding human action control. Apparently, very simple mechanisms that lead to feature binding and retrieval of recently integrated features have an important influence on planning and execution of actions. Regarding the importance of these mechanisms, it seems to be reasonable to test whether they can be measured outside of a formal laboratory situation. Here we ran an online version of the distractor-response binding task reaching participants via crowdsourcing. Distractor-response binding effects were significant in this setup showing that basic mechanisms of feature binding and retrieval indeed influence human action in less formal situations. Besides arguing for the generality and robustness of the effect practical implications are discussed.

## Introduction

Feature-binding is an important mechanism in action control and has increasingly gained interest in recent years (Henson, Eckstein, Waszak, Frings, & Horner, 2014). Carrying out a simple response like a keypress leads to integration of response features with features of the stimuli, present at responding and effect features resulting from the response. Extending the concept of Kahneman and Treisman (1984) object files, integration is assumed to result in an *event file* that includes (binary) bindings between feature pairs (Hommel, 2004; Hommel, Müsseler, Aschersleben, & Prinz, 2001). If any part of the event file is then reencountered later on, other bound parts can be retrieved and influence current responding. Response retrieval due to stimulus repetition, for example leads to response facilitation, if the retrieved and the required response match, but to response impairment, if the retrieved and required responses do not match. According to the *Binding and Retrieval in Action Control* framework (Frings et al., 2020), these core mechanisms of feature integration and retrieval impact behavior observed in various paradigms, used to study human action control (e.g., task switching, negative priming, Posner cueing). Moreover, the same mechanisms might play a role in action related areas like visual search or memory and learning (Frings et al., 2020; Giesen & Rothermund, 2014a). An extensive literature on binding effects has by now identified binding of response features to targets (Hommel, 1998), effects (Dutzi & Hommel, 2009), distractor stimuli (Frings & Rothermund, 2011), tasks (Koch & Allport, 2006), and even other responses (Moeller & Frings, 2019a). Notably, the latter indicate that binding mechanisms seem to be of relevance far beyond the analysis of individual simple responses, but might also play a role in the coordination of complex actions.

Even though binding mechanisms are arguably central in human action control, looking at the vast majority of studies, one might get the impression that binding effects are a phenomenon of the young and well educated. With very few exceptions (e.g., Giesen, Eberhard, & Rothermund, 2015; Giesen, Weissmann, & Rothermund, 2018) the typical sample showing binding effects was recruited at a university and included few participants over 30 years of age. Furthermore, participants were invited into a laboratory and the observed effects typically emerge in a controlled and thus artificial environment. However, if feature binding and retrieval are indeed basic mechanisms in human action control, neither the site of recruitment, nor the situation in which actions are carried out, should be decisive for the mechanisms to influence human performance.

With the current study, we want to take a first step in looking at action control in uncontrolled (i.e., non-laboratory) settings. Specifically, we asked whether it is possible to measure binding effects online. If we find binding effects in an online sample, it would imply that binding effects are generalizable beyond formal laboratory settings, and samples, collected at universities. Moreover, the possibility to measure binding effects online would also facilitate access to groups that have difficulties, coming to a laboratory (e.g., elderly people, people living far from the next university, clinical groups, etc.). In turn, the present results might pave the way to more research regarding, for example cultural, differences in basic mechanisms of human action control. Hence, instead of recruiting students at a university, we ran an online study using crowdsourcing (e.g., Amazon Mechanical Turk) and measured binding between distractor stimuli and responses.

The typical distractor-response binding paradigm implements a prime-probe sequence and in each prime and each probe, participants respond to a target stimulus that is presented together with (oftentimes flanking) distractor stimuli (see Frings & Rothermund, 2011). It is then assumed that distractor stimuli are integrated with the response during the prime, so that repetition of the same distractors in the probe can influence probe performance. Repeating distractor stimuli from the prime as distractor stimuli in the probe then leads to increased performance as compared to distractor changes between prime and probe, if the response has to be repeated. This advantage of distractor repetition is smaller or even turns into a disadvantage, if the response changes between prime and probe. Statistically, distractor-response binding effects thus manifest in an interaction of response relation (from prime to probe) with distractor relation. To anticipate the results, we did indeed find behavior of an online sample to be influenced by binding and retrieval mechanisms.

## Experiment

### Method

#### Participants

Sample sizes in distractor-response binding studies in the laboratory range from less than twenty (e.g., Moeller & Frings, 2011) to more than 80 participants (e.g., Giesen, Frings, & Rothermund, 2012), with many studies drawing around thirty participants (e.g., Moeller & Frings, 2014). Here we decided for a relatively conservative sample size, regarding power, and recruited 54 (33 male) participants. The median age of the sample was 32 years with a range from 21 to 61 years. One participant selected German as their preferred language, the remaining participants of the sample selected English. All participants took part in exchange for monetary reimbursement.

#### Design

The design comprised two within-subjects factors, namely response relation (response repetition vs. response change from prime to probe), and distractor relation (distractor repetition vs. distractor change from prime to probe).

#### Materials

The experiment was conducted using the labvanced platform (https://www.labvanced.com/) which is connected to crowdsourcing platforms with over 7 Million registered participants worldwide and gives Amazon Mechanical Turk, Prolific, Crowdflower, and Clickworker as examples, in addition to an own database. The labvanced system did not restrict participation based on used device. That is, participation was allowed via Computer, smartphone, or tablet. However, all participants in the present sample participated via computer. Instructions were shown in white on black background. The letters D, F, J, and K, presented in red, were used as target- and the letters G and H, presented in green, were used as distractor-stimuli. All individual letters subtended a horizontal and vertical visual angle of 0.7° × 0.7°. A target letter was always presented together with two flanking distractor letters. This stimulus setup subtended a horizontal visual angle of 2.2°. Participants responded to the identity of the target letters by pressing one of two keys on the computer keyboard with their index fingers.

#### Procedure

**Fig. 1:**
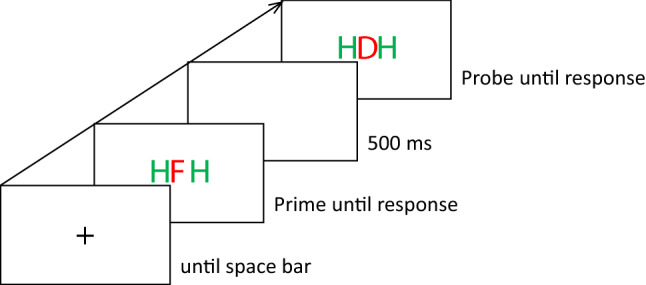
Sequence of events in one example trial. Participants responded to the identity of the central letter by pressing one of two response keys. This is an example for a response repetition/target change and distractor repetition trial. White is depicted in black and black is depicted in white; stimuli are not drawn to scale

Participants first selected English or German as their study language and agreed to recording of personal data and responses during the experiment. Then they calibrated the screen by indicating their distance and adjusting a presented rectangle to the size of a standard ID-card (85.6 × 54 mm). Finally participants indicated their gender, age, and preferred language before the main experiment started. Instructions were given on the screen. Participants were instructed to place their left index finger on the key D and their right index finger on the key K. They were told to respond to the identity of the red and central target letters and to ignore the green and flanking distractor letters. For D and F they were instructed to press the left, and for J and K they were instructed to press the right key. A single trial comprised the following events (see Fig. 1). Participants started each trial by pressing the space bar. Then the prime target- and distractor-stimuli were presented until participants pressed one of the response keys. In case of an incorrect response, a message appeared for 1500 ms, reminding the participant to respond as quickly as possible but without making errors. Then a blanc screen appeared for 500 ms and was followed by the probe target- and distractor-stimuli which again stayed on the screen until participants responded via one of the response keys. In case of an incorrect response, again a message appeared for 1500 ms, reminding the participant to respond as quickly as possible but without making errors. Then a plus sign appeared as a fixation mark, indicating that the next trial could be started.

Response relation between prime and probe (repetition vs. change) was varied orthogonally to distractor relation (repetition vs. change). In response repetition trials (RR), the same response was required to the prime target letter as to the probe target letter. In response change trials (RC), different responses were required to the prime- and to the probe target letter. In distractor repetition trials (DR), the prime distractor letters were repeated as probe distractor letters. And in distractor change trials (DC), prime and probe distractor letters differed. These relations resulted in the four conditions response repetition with distractor repetition (RRDR), response repetition with distractor change (RRDC), response change with distractor repetition (RCDR), and response change with distractor change (RCDC). Response repetition trials were implemented once with target repetition and once with target change between prime and probe. Each of the trial types was realized in 32 trials, resulting in 192 experimental trials. Trial types were assigned first and stimuli were then selected randomly, given that they corresponded to the current response-, target-, and distractor relations. The first 20 trials were treated as practice trials and omitted from the analyses.

### Results

For the analysis of response times (RTs), we considered only those trials with correct responses in both prime and probe. Prime error rate was 3.37%. Probe error was 3.63% (only including trials with correct prime responses). RTs that were more than 1.5 interquartile ranges above the third quartile of the RT distribution of the participant (Tukey, 1977) and RTs that were shorter than 200 ms were excluded from the analysis. Due to these constraints, 13.34% of the trials were excluded for the RT analyses. For mean RTs and error rates, see Table 1.

**Table 1:** Mean response times (in ms) and mean error rates (in percent in parentheses) for probe responses of the current experiment, as a function of response relation and distractor relation

	Response repetition	Response change
Distractor change	620 (2.1)	735 (6.1)
Distractor repetition	606 (1.6)	739 (8.2)
Priming effect	14 (0.5)	− 4 (− 2.1)
Binding effect	18 (2.6)	18 (2.6)

#### Online DRB effects

**Fig. 2:**
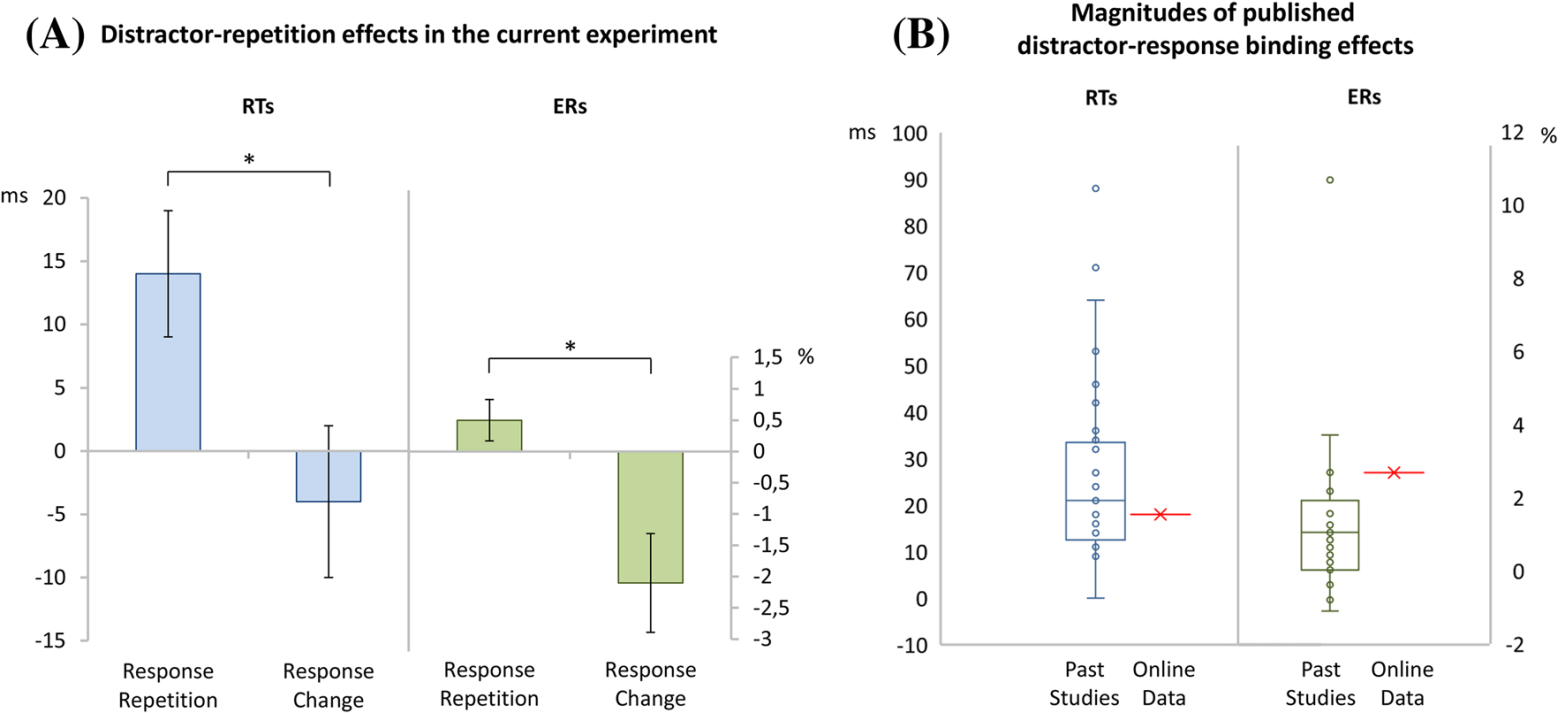
**a** Distractor repetition effects, calculated as distractor change minus distractor repetition trials, as a function of response relation (response repetition vs. response change) for response times and error rates in the current online sample. **b** Distributions of distractor-response binding effect in ms and error rates of 31 experiments in 15 publications (boxplots) as compared to the current distractor response binding effects of the online sample (in red). Binding effects are calculated as the difference between distractor repetition effects in response repetition and response change trials: (Response Repetition/Distractor Change − Response Repetition/Distractor Repetition) − (Response Change/Distractor Change − Response Change/Distractor Repetition) (colour figure online)

In a 2 (response relation: repetition vs. change) × 2 (distractor relation: repetition vs. change) MANOVA on probe RTs with Pillai’s trace as the criterion, the main effect response relation was significant, *F*(1,53) = 138.44, *p* < 0.001, *η*_p_^2^ = 0.72, while the main effect distractor relation was not *F*(1,53) = 1.85, *p* = 0.179, *η*_p_^2^ = 0.03. Participants responded faster if the response was repeated (*M* = 613 ms, SD = 150) than if it changed (*M* = 737 ms, SD = 193), between prime and probe. More importantly, the interaction of response relation and distractor relation was significant as well, *F*(1,53) = 4.24, *p* = 0.044, *η*_p_^2^ = 0.07 (see, Fig. 2a, left hand side), indicating binding between distractor stimuli and responses.^1^ Distractor repetition facilitated performance only if the response was repeated, as well.

In the same analysis on error rates, the main effect of response relation was significant, *F*(1,53) = 47.68, *p* < 0.001, *η*_p_^2^ = 0.47, while the main effect of distractor relation was not, *F*(1,53) = 2.53, *p* = 0.118, *η*_p_^2^ = 0.05. Importantly, the interaction of response relation and distractor relation was again significant, *F*(1,53) = 7.05, *p* = 0.010, *η*_p_^2^ = 0.12 (Fig. 2a, left hand side). That is, we found clear indication of distractor-response binding also in the error rates.

#### Comparison with previous laboratory DRB effects

To rate the magnitudes of the present binding effects before the background of previous work, we selected 15 studies investigating distractor-response binding effects that used a similar visual setup as used in the present experiment (see Table 2). These reported 31 experiments with data to calculate the magnitudes of distractor-response binding effects in RTs and/or in error rates. Binding effects were calculated as the difference between distractor repetition effects in response repetition and response change trials ([Response Repetition/Distractor Change − Response Repetition/Distractor Repetition] − [Response Change/Distractor Change − Response Change/Distractor Repetition]). Across these studies, distractor-response binding effects had a mean magnitude of 27 ms and 1.3% errors. The present effects of 18 ms and 2.6% errors did not significantly differ from these means, *t*(53) = 1.16, *p* = 0.252, *d* = 0.16 for RTs, and *t*(53) = 1.38, *p* = 0.172, *d* = 0.19 for error rates. A Bayes factor of BF_01_ = 3.576 (RTs; calculated via JASP) indicated that the data are three times more likely under the null hypothesis that postulates identical binding effects than under the alternative hypothesis that postulates a difference between binding effects (Wagenmakers et al., 2018). The same analyses for error rates resulted in BF_01_ = 2.747, which is indecisive. Yet, note that any difference between past binding effects in error rates and the current effect would indicate a larger effect in the current online sample. See Fig. 2b for a visualization of the present magnitudes as compared to magnitudes in past studies.

**Table 2:** Past publications reporting a distractor-response binding effect (DRB) in response times (in ms) and/or error rates (in percent)

Publication	DRB (ms)	DRB in % errors
Frings (2011)	24	
Frings and Moeller (2010)		
Exp 1	11	0.2
Exp 2	10	1.2
Frings and Moeller (2012)	9	0.8
Frings, Moeller, and Rothermund (2013)		
Exp 1	88	1.1
Exp 2	25	0.7
Frings, Rothermund, and Wentura (2007)		
Exp 1a	53	1.6
Exp 1b	27	− 0.8
Exp 2	27	1.3
Giesen et al. (2015)		
18–27 years	46	1.2
60–64 years	64	0.0
65–78 years	42	− 1.1
Giesen et al. (2012)		
Exp 1a	21	− 0.75
Exp 1b	12	1.5
Exp 2	10	0.0
Giesen and Rothermund (2011)	16	2.25
Giesen and Rothermund (2014a)		
Exp 1	9.5	− 0.8
Exp 3	12	0.6
Giesen and Rothermund (2014b)		
Exp 2	27	
Exp 3	22	
Giesen and Rothermund (2015)		
Pos. contingency	71	10.4
Neg. contingency	19	2.6
Orthogonal	32	3.6
Laub, Frings, and Moeller (2018)		
Exp 1	15	2.1
Exp 2	14	0.7
Moeller and Frings (2014)		
Exp 1	18	2.1
Exp 2	15	2.2
Exp 4	34	0.4
Moeller and Frings (2017a)		
Exp 1	21	1.0
Exp 2	15	1.3
Moeller and Frings (2017b)		
Non-words	36	− 0.4

## Discussion

We measured distractor-response binding effects in participants that were recruited via crowdsourcing online, participated remotely (i.e., not at a laboratory) and showed a much larger variety in age than the typical sample of university students, of most previous studies. Notably also with this difference in setting and for this somewhat different sample, the standard distractor-response binding effects were observed. That is, distractor-response binding effects are indeed generalizable to an online sample, meaning that binding- and retrieval mechanisms impact human behavior also outside of formal laboratory settings, and beyond samples, collected at universities. This result opens new possibilities for research on human action control in groups, difficult to access in a way that has been conventional for the last decades (i.e., inviting participants into a university’s laboratory). Particular groups for which this might be relevant are clinical samples and less mobile or rural groups. Similarly, being able to measure mechanisms in action control online also facilitates cross-cultural comparisons.

Even though we measured binding between distractor stimuli and responses, it should be noted that binding mechanisms seem to function identically, independent of the origin (e.g., stimulus or response) of the encoded features. This is in line with the common coding assumption (Prinz, 1992), which lies at the heart of binding mechanisms: Representations of stimuli and representations of responses are encoded in one system so that codes of perception and codes of action do not differ and can directly overlap (Hommel, 2009). Various empirical evidence supports the common coding assumption. For example, distractor-response and response-effect binding effects correlate and are modulated identically by response pacing (Moeller, Pfister, Kunde, & Frings, 2016). Also different sorts of bindings follow the same assumptions regarding a binary quality, meaning that independent of their original order, repeating one feature can retrieve the other (see, Hommel, 2004): evidence for this quality exists in studies targeting response-effect and also response-response binding (Dutzi & Hommel, 2009; Moeller & Frings, 2019b, c). Taken together, slightly differently measured (response–effect-, stimulus–response-, response–response-, etc.) binding effects can be assumed to rely on identical processes, and it seems safe to assume that our present findings not only apply to distractor-response binding effects, but that binding mechanisms in general are relevant and measurable in a population accessible online.

Intriguingly, evidence that binding mechanisms influence behavior that is measurable online also has a very direct implication for the practical design of websites: It means that these mechanisms may impact click choices online. In the present study, correct responses were predefined and binding- and retrieval mechanisms manifested in error rates and response times. In an online scenario, this means that repeated encounters of salient visualizations have the potential to retrieve former actions, which might lead to errors while interacting with a website. Maybe even more relevant, the same mechanisms can influence choices in situations, where responses cannot be labelled “correct” or “incorrect” in advance (Dutzi & Hommel, 2009; Moeller et al., 2016, 2019). That is, choices in an online interaction might be tipped in the direction of repeated or changed responses depending on whether or not salient stimuli from before are presented again.

Taken together, binding mechanisms seem to play a role in action control of a more general population than previously tested. Specifically, a population that is accessible online shows the same binding effects as previously reported mostly for samples of university students. This is an important piece of information, if online (click-) action is of interest. It also underlines the generalizability of binding mechanisms and opens new possibilities to compare groups of participants that have been difficult to access in laboratory studies.

## Footnotes

1 Contrasts in a 3 (response relation: response repetition with target repetition vs. response repetition with target change vs. response change) × 2 (distractor relation: repetition vs. change) analyses indicated that this interaction was not due to differences between target repetition and target change trials *F*(1,53) < 1, *p* = 0.468, *η*_p_^2^ = 0.01, but rather due to a difference between response repetition and response change trials, *F*(1,53) = 2.91, *p* =0 .047 (one sided), *η*_p_^2^ = 0.05.
